# A pH-Sensitive Lignin-Based Material for Sustained Release of 8-Hydroxyquinoline

**DOI:** 10.3390/polym15081867

**Published:** 2023-04-13

**Authors:** Qian Zheng, Lanfang Chai, Boyu Du, Wei Li, Lian-Hua Fu, Xiaohong Chen

**Affiliations:** 1 Liaoning Key Laboratory of Lignocellulose Chemistry and Biomaterials, Dalian Polytechnic University, Dalian 116034, China; z1424072039@163.com (Q.Z.);; 2 School of Biomedical Engineering, Shenzhen University, Shenzhen 518060, China

**Keywords:** pH-sensitive, lignin-based polymer, 8-hydroxyquinoline

## Abstract

The fabrication of pH-sensitive lignin-based materials has received considerable attention in various fields, such as biomass refining, pharmaceuticals, and detecting techniques. However, the pH-sensitive mechanism of these materials is usually depending on the hydroxyl or carboxyl content in the lignin structure, which hinders the further development of these smart materials. Here, a pH-sensitive lignin-based polymer with a novel pH-sensitive mechanism was constructed by establishing ester bonds between lignin and the active molecular 8-hydroxyquinoline (8HQ). The structure of the produced pH-sensitive lignin-based polymer was comprehensively characterized. The substituted degree of 8HQ was tested up to 46.6% sensitivity, and the sustained release performance of 8HQ was confirmed by the dialysis method, the sensitivity of which was found to be 60 times slower compared with the physical mixed sample. Moreover, the obtained pH-sensitive lignin-based polymer showed an excellent pH sensitivity, and the released amount of 8HQ under an alkaline condition (pH = 8) was obviously higher than that under an acidic condition (pH = 3 and 5). This work provides a new paradigm for the high-value utilization of lignin and a theory guidance for the fabrication of novel pH-sensitive lignin-based polymers.

## 1. Introduction

As the second abundant biomass, lignin, which consists of sinapyl alcohols (S-units), coniferyl alcohols (G-units), and p-coumaryl alcohols (H-units), has attracted great research interest in the development of renewable and low-cost materials. Among the numerous research orientations, the development of lignin-based smart materials, such as pH-, temperature-, and light-responsive polymers, is one of the most potential ways to realize the high-value utilization of lignin. As their name implies, pH-responsive lignin-based polymers will marvelously change their shape and/or properties in response to an external pH stimulation. Generally, the pH-sensitive property mainly depends on the ionization and protonation of the oxygen-containing groups in a lignin structure, for example, the hydroxyl groups. However, the hydroxyl groups are usually served as the reactive sites for fabrication of the lignin-based polymer. Therefore, there is a contradiction since these hydroxyl groups will obviously decrease if other chemical skeletons are introduced by covalent bonds, which impairs the pH-responsiveness. However, if these oxygen-containing groups are retained, the other functions of the fabricated lignin-based product would be unsatisfied. In the previous work, we made an effort to ease this conflict by increasing the hydroxyl content via demethylation. However, this did not solve the problem at its root. Therefore, it is urgent to develop novel lignin-based polymers with a different pH-responsive mechanism.

Although the research on lignin-based pH-responsive polymers is still in its infancy, other types of pH-sensitive conjugates with different responsive mechanisms are relatively mature. For instance, a polymer fabricated with acid-unstable bonds, such as ester bonds [1,2,3], hydrazones [4,5], imines [6,7,8], phosphoramidates [9], acetals [10,11], cyclic acetals [12], and β-thiopropionate bonds [13,14,15], can realize pH response dissociation via the breaking of the weakly covalent bonds. Obviously, the ester bonds can be easily constructed in lignin by a simple esterification reaction. In addition, ester bonds can also be broken under alkaline conditions, which expands the utilization potential of the corresponding polymers [16]. Therefore, the introduction of ester bonds to synthesize a novel lignin-based polymer is expected to be a feasible way to resolve the aforementioned problem.

8HQ is a good monophasic bidentate chelating agent, which can form four- and six-covalent complexes with a wide range of metal ions, including Cu^2+^, Zn^2+^, Bi^2+^, Mn^2+^, Mg^2+^, Cd^2+^, Ni^2+^, Fe^3+^, and Al^3+^ [17,18,19,20,21]. Based on this, 8HQ has typically been usually used for corrosion protection of metal [22,23] and the examination and separation of metal ions. For example, Crespy and coworkers [23] prepared pH-sensitive polymer conjugates from 8HQ, ethyl acrylate, and 2-mercaptoethanol, which could well be utilized in the corrosion protection of metal. Additionally, it has a broad prospect in pharmaceutical engineering [24,25,26,27,28,29,30]. Kljun et al. [25] demonstrated the anticancer potential of organ ruthenium complexes of 8HQ. Chauhan et al. [26] prepared a therapeutic agent for botulinum neurotoxins from 8HQ. Buchler et al. [27] optimized the inhibitory effect of 8HQ inhibitors on catechol O-methyltransferase. Pippi et al. [28] elucidated the mechanism of antifungal action of 8-hydroxyquinolines. Odingo and Early et al. [29] demonstrated the bactericidal effect of 8-hydroxyquinolines on Mycobacterium tuberculosis. Therefore, combining the above-mentioned significance of the utilization of lignin, the preparation of 8HQ-contained lignin-based polymers might be very important. Herein, a pH-responsive lignin-based polymer was prepared by constructing ester bonds between lignin, 8-Hydroxyquinoline (8HQ), and adipoyl chloride. As compared with the physical blending sample, the conjugate of lignin with 8HQ shows about a 60 times slower release rate. The prepared lignin-based polymers also showed a great pH-sensitive 8HQ release performance, which demonstrates great potential for the removal of heavy metal ions from wastewater, corrosion prevention of metal materials, and development in the pharmaceutical field.

## 2. Materials and Methods

### 2.1. Materials

The lignin (LL) used in this study was obtained from Shanghai Dongsheng New Materials Co., Ltd., Shanghai, China, and was purified by dialysis in a 300 molecular weight dialysis bag before modification. Adipoyl chloride, 8-hydroxyquinoline (8HQ), sodium carbonate (Na_2_CO_3_), sodium hydroxide (NaOH), triethylamine (Et_3_N), N, N-dimethylformamide (DMF), tetrahydrofuran (THF), hydrochloric acid (HCl), pyridine (Py), acetic anhydride (AA), pentafluorobenzaldehyde, deuterate dimethyl sulfoxide (DMSO-d6), and potassium bromide (KBr) were purchased from Energy Chemical. All the chemicals were used without further purification.

### 2.2. Synthesis of a pH-Responsive Lignin-Based Polymer

**Scheme 1:**
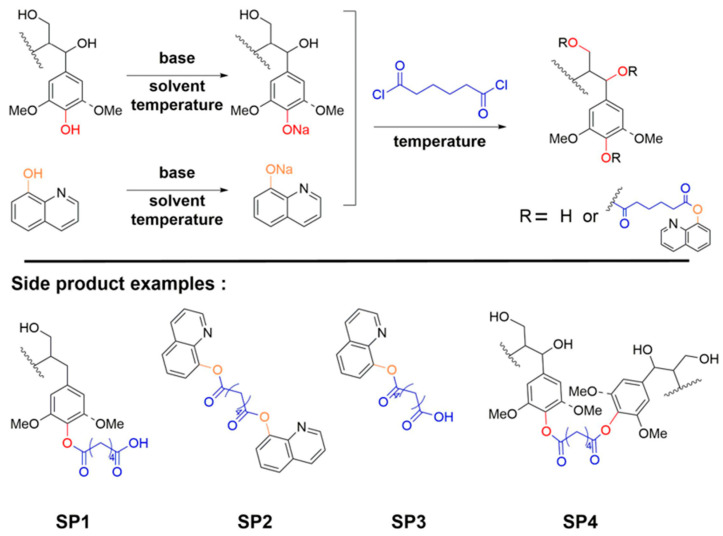
The procedure for the preparation of a pH-responsive lignin-based polymer.

The general procedure for the preparation of a pH-responsive lignin-based polymer is shown in Scheme 1. Briefly, in a 100 mL bottle, lignin (1.0 g), 8HQ (1.31 g) and base (0.45 g) were dissolved in the organic solvent. The resultant mixture was stirred at 60 °C for 30 min. After cooling the mixture to room temperature, adipoyl chloride (2.14 g) was added drop by drop. Subsequently, the temperature was increased to 60 °C. After 10 h, the reaction was precipitated with dilute hydrochloric acid (1M). Finally, the precipitate was washed and lyophilized to obtain the product (L-60) as a brown solid. All the other samples were prepared based on the reaction conditions of L-60, but one of the variables was altered. L-40 and L-80 are samples in which the temperature was change to 40 and 80 °C, respectively. L-Et and L-Na indicate that the alkaline additive was changed to Et_3_N and Na_2_CO_3_, respectively. L-THF signifies that the solvent was changed to THF. The detailed reaction conditions of each sample are summarized in Table 1.

### 2.3. Acetylation of the pH-Responsive Lignin-Based Polymer

Firstly, 200 mg of the lignin-based polymer was dissolved in 4 mL of pyridine to form a homogeneous solution. Then, 4 mL of acetic anhydride was added to the mixture, and the mixture was stirred at room temperature for 72 h. After that, cold deionized water was added dropwise to form a precipitate. After washing and freeze-drying, the acetylated lignin sample was obtained as a black solid.

### 2.4. Calculation of the Content of 8-Hydroxyquinoline

The lignin-based polymer (5 mg) was added to a sodium hydroxide solution (pH = 11, 200 mL). Then, the reaction was heated to 80 °C. After stirring for 4 h, the content of 8HQ in the solution was determined by UV at 253 nm. Finally, the weight percentage of 8HQ was calculated based on the pre-determined standard curve.

### 2.5. Release of 8HQ from the Polymer

The λ_max_ of 8HQ in citrate buffer (pH = 3 and 5) and phosphate buffer (pH = 7 and 8) was determined to be 249, 248, 239, and 239 nm, respectively. The calibration curves of the content of 8HQ at four different pH values were determined using λ_max_. These calibration curves were then used to calculate the amount of 8HQ released at different time intervals. Firstly, 5, 10, 15, and 20 mg of the sample were separately placed in dialysis bags (molecular weight cutoff = 300 Da). Then, the dialysis bags were immersed in 200 mL of a buffer medium with pH values of 3, 5, 7, and 8. At different time intervals, 10 mL of the buffer medium outside the bag was removed for ultraviolet (UV) detection at λ_max_ and was replaced with 10 mL of fresh buffer solution to keep the volume constant. This process lasted for 8 days. For investigating the slow-release effect of the produced polymer, a physical mixed sample (PM, 5 mg) with a mass ratio of 77:23 (lignin to 8HQ) was set up as a control group. The mass ratio of PM was confirmed by the substitution degree (SD) of 8-hydroxyquinoline in L-Na, the sample that was used to analyse the release performance. In order to discuss the impact of side-products, the L-Na was dialyzed with distilled water in a dialysis bag (molecular weight cutoff = 1000 Da) for 72 h to produce the purified polymer PL-Na. Then, the experiment of the release of 8HQ was conducted using PL-Na as the sample.

### 2.6. Characterization

#### 2.6.1. Ultraviolet Spectrophotometer (UV)

The ultraviolet spectrometer absorption spectra were measured with an ultraviolet spectrophotometer (Cary 300, Agilent, Santa Clara, CA, USA).

#### 2.6.2. Gel Permeation Chromatography (GPC)

The molecular weights of the lignin and pH-responsive lignin-based polymers were measured by gel permeation chromatography (GPC, Agilent 1200, Santa Clara, CA, USA) equipped with a PL-gel 10 mixed-B 7.5 column. The mobile phase is THF with a speed of 1.0 mL/min.

#### 2.6.3. Fourier Transformation Infrared Spectroscopy (FT-IR)

The FT-IR spectra of the lignin and pH-responsive lignin-based polymers were performed by a PerKin-Elmer spectrophotometer, Waltham, MA, USA. The tablets were prepared by grinding the sample and KBr with a mass ratio of 1:100. The scanning frequency ranges from 4000 to 500 cm^−1^ with a resolution of 1 cm^−1^ in the transmission mode.

#### 2.6.4. Thermogravimetric Analysis (TGA)

The thermal stability was detected via thermos gravimetric analysis (TGA; TA Instruments Q500, New Castle, DE, USA). The 6.0 mg sample was placed in a small crucible under a nitrogen atmosphere. The temperature was increased from 25 °C to 700 °C with a heating rate of 10 °C/min.

#### 2.6.5. Surface Wettability

The wettability of the sample surfaces was tested by the seated-drop method using a contact-angle tester (LSA100, LAUDA Scientific, Lauda-Königshofen, Germany). The static contact angle (CA) of the buffered solutions (citric acid-sodium citrate buffer solution, pH = 3 and 5; phosphate buffer solution, pH = 7 and 8)/solid powder was tested with a droplet size of 3 mm.

#### 2.6.6. Nuclear Magnetic Resonance (NMR) Spectra

The ^1^H NMR and 2D-heteronuclear single quantum coherence (2D-HSQC) spectra were recorded on a Bruker Avance-400 MHz spectrometer 193 instrument (Bruker, Billerica, MA, USA). For ^1^H NMR, the sample (25 mg), pentafluorobenzaldehyde (15.88 mg), and DMSO-d_6_ (0.55 mL) were added to a nuclear tube. Each spectrum was scanned 64 times. For 2D-HSQC, 50 mg of lignin or lignin-based polymer was dissolved in 0.55 mL of DMSO-d_6_, and the resultant mixture was scanned 64 times. The hydroxyl contents were calculated according to Equations (1)–(3):

(1) $$K\left(Ar\right)\;\left(\mathrm{mmol}/g\right)=\frac{m_{1}\times S_{2}}{3\times M_{1}\times S_{1}\times m}$$

(2) $$K\left(Al\right)\;\left(\mathrm{mmol}/g\right)=\frac{m_{1}\times S_{3}}{3\times M_{1}\times S_{1}\times m}$$

(3) $$K\left(Tot\right)\;\left(\mathrm{mmol}/g\right)=K\left(Ar\right)+K\left(Al\right)$$

where *K(Ar)* is the content of phenolic hydroxyl groups; *K(Al)* is the content of aliphatic hydroxyl groups; *K(Tot)* is the content of total hydroxyl groups; *m*_1_, *S*_1_, and *M*_1_ are the mass, the integration of the resonance peak, and the molar molecular mass of pentafluorobenzaldehyde, respectively; *S*_2_ and *S*_3_ are the integration of the aromatic and aliphatic acetoxy groups, respectively; and m is the mass of the lignin or lignin-based polymer.

## 3. Results and Discussion

### 3.1. The Structure of Lignin

**Figure 1:**
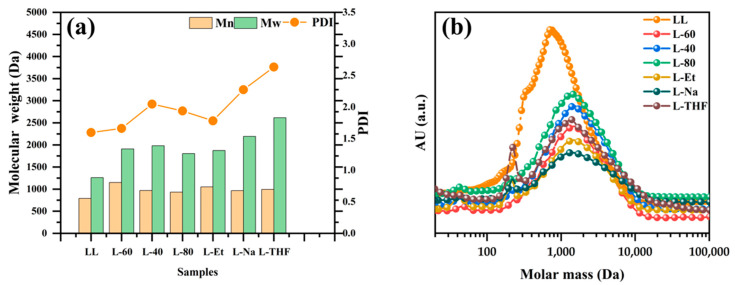
The molecular weights (**a**) and distributions (**b**) of lignin and pH-responsive lignin-based polymers.

The weight-average (M_w_) and number-average (M_n_) molecular weights of the eight samples were determined by GPC analysis, and the corresponding polydispersity indexes (PDI) were calculated. The results are shown in Figure 1. As can be seen, as compared with the original lignin LL, the M_w_ and M_n_ of all the prepared lignin-based polymers increased. Especially in the samples of L-Na and L-THF, about two times the M_w_ was obtained, indicating that the 8HQ and adipoyl chloride might be introduced into the structure of lignin through ester bonds. However, the PDI of these samples were also higher than the others, suggesting that the produced lignin-based polymers are more heterogeneous. One of the reasons for this outcome might be the formation of side products. Another reason might be the polycondensation of lignin during the ester modification.

**Figure 2:**
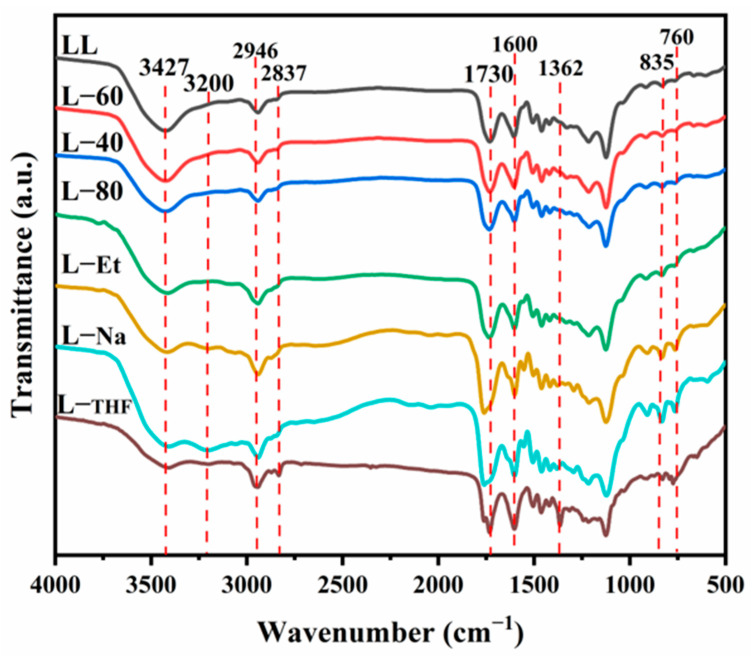
The FT-IR spectra of lignin and pH-responsive lignin-based polymers.

The FT-IR spectra of lignin and lignin-based polymers are shown in Figure 2. The curve of LL is consistent with the characteristic absorption bands of molecular lignin, including the stretching vibration absorption bands of the hydroxyl group (3427 cm^−1^), C-H bond (2946 and 2837 cm^−1^), and the skeleton vibration of the aromatic ring (1600–1400 cm^−1^). As compared with LL, in the curves of the all-lignin-based polymers, especially the samples of L-Et, L-Na, and L-THF, the characteristic bands at 1730 cm^−1^ attributed to the C=O bond are stronger, and the bands at 3427 cm^−1^ are apparently weaker, indicating the successful introduction of ester groups. Moreover, the bands at 3200 cm^−1^ in the curves of the prepared lignin-based polymers assigned to the unsaturated C-H bonds obviously increased, suggesting that the depolymerization might occur during the modification. Moreover, the absorption strength of the bands at 1362 cm^−1^ due to the deformation vibration of the CH_3_COO group and the bands at 835 and 760 cm^−1^ attributed to the deformation vibration of the substituted benzene ring are higher after esterification, indicating that the 8HQ might be grafted to the lignin through ester bonds.

**Figure 3:**
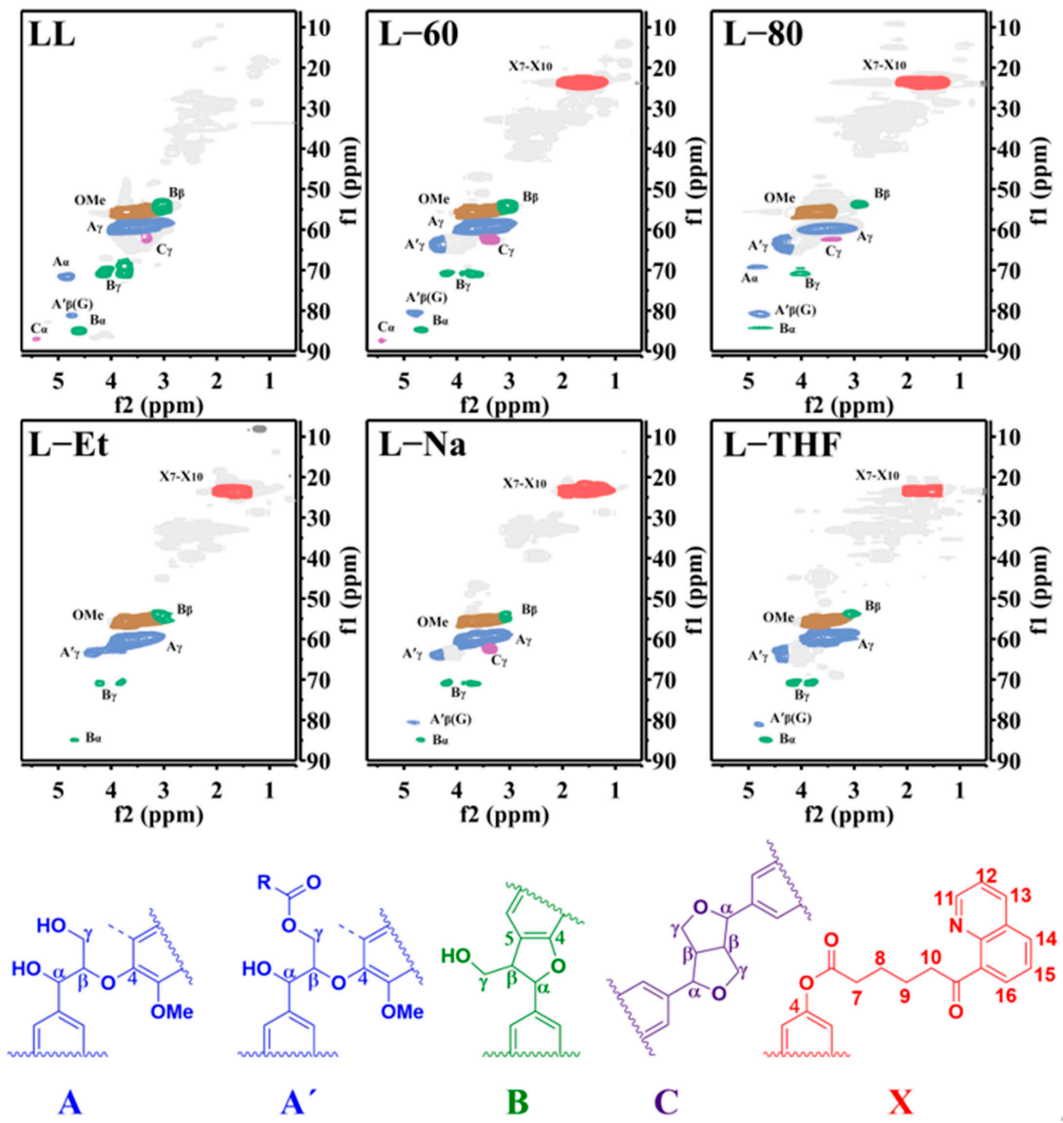
The aliphatic side-chain regions of the 2D-HSQC NMR spectra of LL, L-60, L-80, L-Et, L-Na, and L-THF.

**Figure 4:**
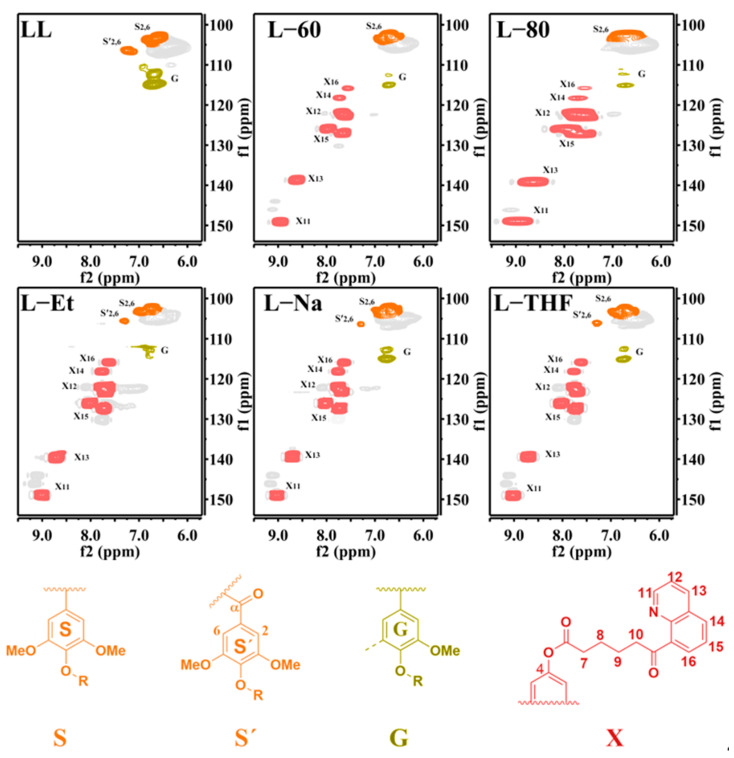
The aromatic regions of 2D-HSQC NMR spectra of LL, L-60, L-80, L-Et, L-Na and L-THF.

In order to further understand the structural change of lignin during esterification, a 2D-HSQC analysis was conducted. As shown in Figure 3, in the regions of aliphatic side chains, the peaks of Aα (δH/δC = 4.86/71.87 ppm), A′β(G) (δH/δC = 4.75/81.1 ppm), Aγ (δH/δC = 3.64/59.5 ppm), and A’γ (δH/δC = 3.74/61.9 ppm) appear in the spectrum of LL, but no signal of Aβ(G), Aβ(S), or A’β(S) is found, indicating that the content of β-O-4 linkages is low in the original sample. The signals of β-5 linkages Bα (δH/δC = 4.61/85.0 ppm), Bβ (δH/δC = 3.07/53.6 ppm), and Bγ (δH/δC = 3.75–4.12/70.7 ppm) are clearly observed. The integral of A′γ significantly increased in the samples of L-60, L-80, L-Et, L-Na, and L-THF, indicating that the aliphatic hydroxyl groups are also the active sites for esterification. Importantly, the appearance of the signals at δH/δC = 0.7–2.3/19.94–25.19 ppm, which is attributed to the X_7_–X_10_, demonstrates the successful introduction of adipic acyl. The aromatic regions of the 2D-HSQC spectra are shown in Figure 4. As can be seen, only S_2,6_, S’_2,6_, G_2_, and G_5_ are found in the sample of LL, suggesting that the H unit is absent in the original lignin, and condensation might occur. Moreover, in the polymer samples, the signals of X_11_–X_16_ were detected. Combined with the outcomes of Figure 3, it can be deduced that the target lignin-based polymers were successfully synthesized.

### 3.2. The Substitution Degree (SD) of 8HQ

**Figure 5:**
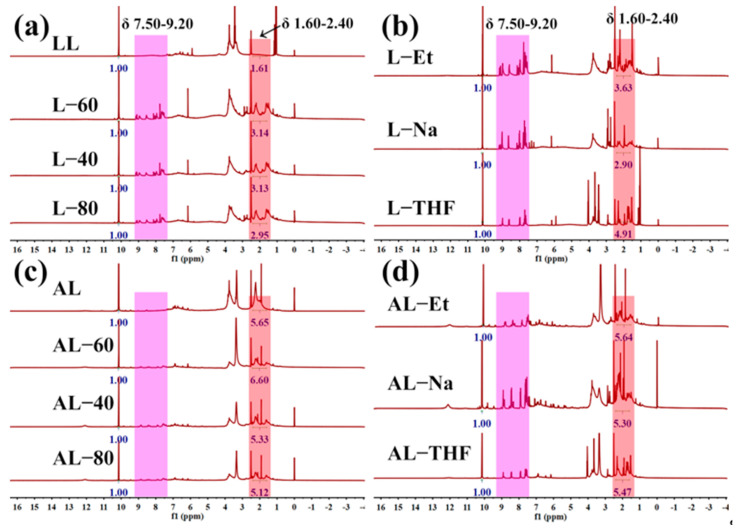
^1^H NMR of lignin samples before (**a**,**b**) and after (**c**,**d**) acetylation.

The decrease of the hydroxyl content was calculated according the ^1^H NMR spectra, and the substitution degree of 8HQ was tested by an alkaline hydrolysis method. In Figure 5, depending on the integral of the acetyl group (δ 1.60–2.40 ppm) in the LL or polymers before and after acetylation, the hydroxyl content could be calculated, and the results are shown in Table 2. As can be seen, the decreases of the hydroxyl content ranged from 14.0 to 86.0%, while the substitution degrees of 8HQ are from 22.7 to 46.6%. That is to say, the side reactions were occurring during the preparation of the target product. For example, in the sample of L-Et, 48.8% of the hydroxyl was decreased, but only 33.0% SD of 8HQ was obtained, suggesting that some of the -OH might be consumed to form the side products, such as SP1 and SP4 (Scheme 1). There was no 8HQ was introduced to these two side products, resulting in the SD being lower than the percentage of the decrease of hydroxyl. The contrast example is L-Na: 39.5% of the hydroxyl was reacted, but 46.6% SD was obtained, indicating that the side-products SP2 and SP3 might be mixed (Scheme 1). In the SP2 and SP3 side-products, the contents of 8HQ are obviously higher than that of the target polymer, leading to the SD being larger than the decrease amount of hydroxyl. After purification via dialysis, the SD of 8HQ in the PL-Na sample was decrease to 34.7%, suggesting that some other unexpected side products containing hydroxyl groups might also exist in the polymer. Additionally, the characteristic peaks of 8HQ (δ 7.50–9.20 ppm) were determined in the polymer samples, which proved the objective products might have been fabricated smoothly.

### 3.3. Thermal Stability of Samples

**Figure 6:**
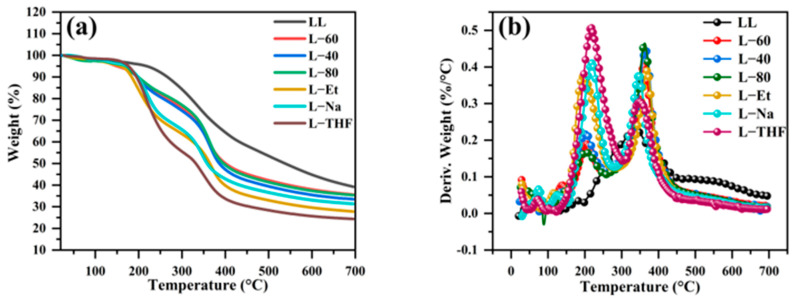
The TGA curves (**a**) and DTA curves (**b**) of lignin and pH-responsive lignin-based polymers.

The thermal stability of the samples was detected by TGA analysis. As shown in Figure 6a, the weight losses of all the samples involved three stages. The first stage from 50 to 150 °C might be attributed to the evaporation of moisture. Then, the fractures of cross-linkages and the degradation of lignin might be the reasons for the mass losses from 150 °C to 300 °C, such as the breaking of α-O-4 linkages and decarboxylation reactions. As can be seen, the weights of all lignin-based polymers were lower than that of LL, especially the samples of L-Et, L-Na, and L-THF. Moreover, the sharp losses ranging from 300 to 500 °C might be due to the cracking of various and functional groups substituted on the lignin units, such as phenolic hydroxyl groups and carbonyl groups. Obviously, the weight of the polymers is lower than that of LL. That is to say, more cross-linkages and more functional groups existed in the polymers, indicating the success of the graft reaction. Finally, the minor loss from 500 to 700 °C might be related to the cleavage of aromatic rings. The char yields of LL, L-60, L-40, L-80, L-Et, L-Na, and L-THF are 39.26%, 35.61%, 33.48%, 35.38%, 36.77%, 27.79%, 31.34%, and 24.34% at 700 °C, respectively. Figure 6b shows the TG derivative curve of all the samples, which implies the rate of mass loss during the pyrogenic decomposition. As can be seen, except the LL, all the produced polymers decomposed quickly under about 200 and 350 °C, which might be due to the breakage of aryl ether bonds and the cracking of phenol groups, etc. Particularly, LL has no peaks around 200 °C in Figure 6b, which might be attributed to the low content of aryl ether bonds. This result is in consistent with the NMR characterization. The degradation rate of LL is higher than all of the other samples in the range of 500–700 °C, suggesting that the content of benzene was decreased due to the graft. In short, the decreasing thermal stability demonstrates successful esterification.

### 3.4. pH-Responsive Switchable Wettability of L-Na

**Figure 7:**
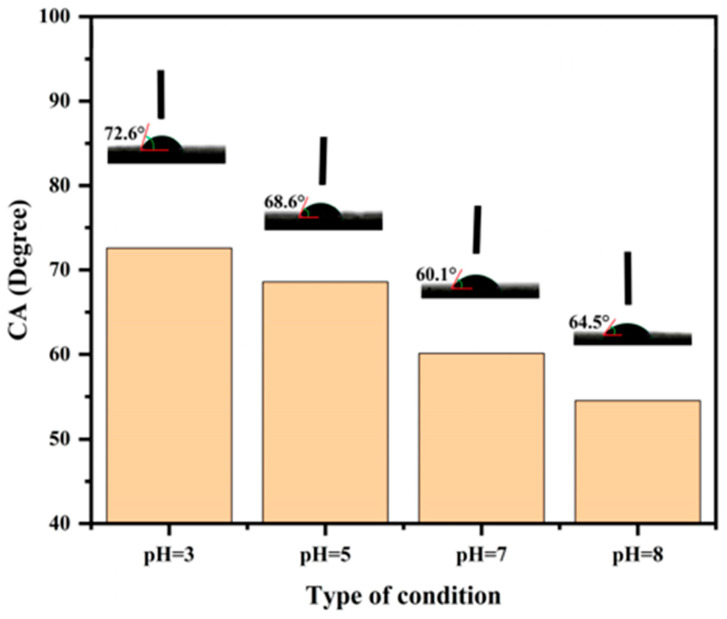
pH-responsive wettability transition of L-Na.3.5. Release of 8HQ.

In order to understand the pH-responsive performance, the switchable wettability of L-Na was detected. As shown in Figure 7, L-Na exhibited very different wettability when placed in different pH environments. The wettability (CA = 60.1°, 54.5°) in an alkaline environment (PH = 7, 8) was better than that in an acidic environment (PH = 3, 5) (CA = 72.6°, 68.6°), and the higher the alkalinity, the better the wettability, which fully demonstrates the pH-responsive property of L-Na.

**Figure 8:**
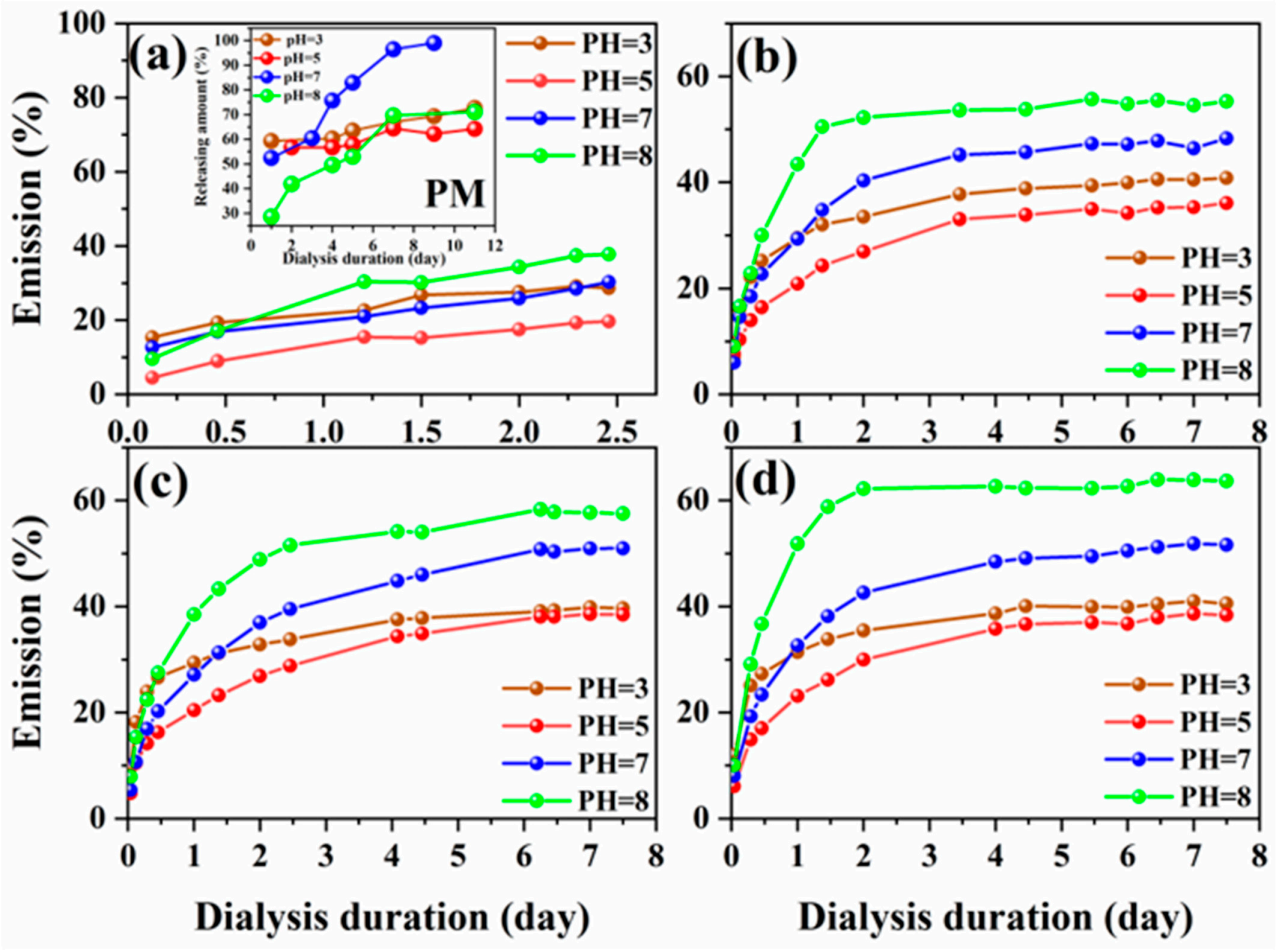
The release performances of 8HQ for (**a**) 5 mg, (**b**) 10 mg, (**c**) 15 mg, and (**d**) 20 mg of pH-responsive lignin-based polymer.

Based on the characterization results above, the SD of 8HQ in the sample of L-Na is the highest. Therefore, L-Na was chosen as the optimal sample for the release experiments. The dosage of L-Na was 5 mg, 10 mg, 15 mg, or 20 mg, and the pH condition was 3, 5, 7, or 8, respectively (Figure 8). Moreover, 5 mg of PM was set as the control sample. As shown in Figure 7a, 99% of the 8HQ in PM can be released in only 9 h at pH = 7. In the acidic conditions (pH = 3 and 5), the release rate was similar to that in a neutral solution initially, but significantly decreased after 1 h. The total release percentages are 72% and 64%, respectively. However, in the base solution (pH = 8), the release rate underwent a slow–fast–slow process. These differences might be because the carboxyl groups in the lignin structure could be esterified by the mixed 8HQ in acidic and alkaline conditions. In contrast, the synthesized pH-responsive lignin-based polymers showed a much slower release rate in not only acidic, but also alkaline conditions. It only released 19.6–37.7% of the 8HQ to the solution, even after 2.5 days. The reason might be that the release of 8HQ requires the breakage of the chemical bonds (ester bonds), which limits the dialysis of 8HQ. That is to say, the slow release of 8HQ could indeed be realized by the strategy proposed by this work, and L-Na showed about 60 times slower release performance than the PM. Figure 6b–d shows the relationship between the emission amount and the time when the dosage of L-Na was increased to 10, 15, and 20 mg. The corresponding releasing amounts of 8HQ increased quickly in the initial 2 days, and then slowly reached 36.1–55.3%, 38.5–57.6%, and 38.4–63.7% (7.5 days), respectively. Obviously, the total emission amount is closely related to the dosage of the samples. Moreover, the releasing percentage under an alkaline condition (pH = 8) is remarkably higher than that under acidic conditions (pH = 3 and 5). This might be because the lignin-based polymer is soluble in an alkaline buffer solution, which could facilitate the cracking of the ester bonds. While in acidic conditions, L-Na was suspended in the buffer solution, resulting in a heterogeneous hydrolysis reaction. Notably, the releasing amount of 8HQ under pH = 3 is higher than that under pH = 5. This might be because the ester groups could undergo hydrolysis easier under more acidic conditions. On the other hand, the formation of quinolinium salts might facilitate the dissociation equilibrium of L-Na shifting to the right for producing 8HQ, which results in a higher amount of 8HQ being released at pH = 3 as compared with pH = 5. In short, the L-Na showed a pH-sensitivity for releasing 8HQ.

**Figure 9:**
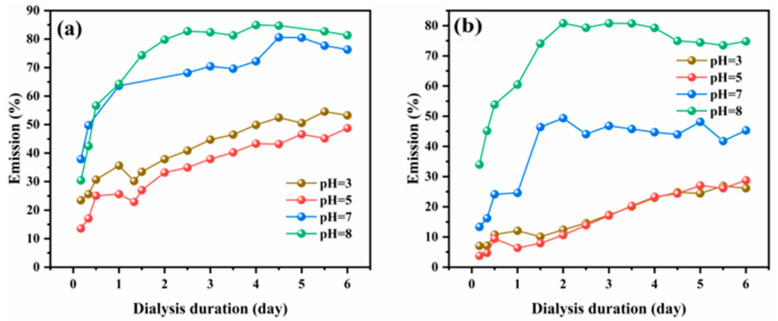
The release performances of 8HQ for (**a**) 5 mg pL-Na and (**b**) 5 mg pL-80.

As the results above show, there are some side-products in L-Na that might influence the release effect of 8HQ. Therefore, the release performance of the purified sample PL-Na was analysed. As shown in Figure 9a, after purification, the release amounts of 8HQ under all the tested pH levels were increased obviously as compared with the L-Na. Moreover, the increase rate of the release amount under alkaline-neutral (pH = 8 and 7) conditions became higher as compared to the acidic (pH = 3 and 5) conditions. Additionally, the difference between the release amount under an alkaline condition and that under an acidic condition increased significantly. The reason might be that the mass percentage of 8HQ in the side-products was lower than that in the purified PL-Na. Furthermore, the release performance of purified L-80 (PL-80) was also examined (see Figure 9b). As can be seen, after 2 days, the percentage of the released 8HQ under an alkaline condition (pH = 8) was remarkably higher than that of the PL-Na, which suggests that the sustained release performance of PL-Na was better.

## 4. Conclusions

In summary, a pH-responsive lignin-based polymer with a novel sensitive mechanism was prepared from lignin, adipic acid chloride, and 8HQ by esterification. The structure of the produced polymer was comprehensively characterized. The GPC results show that the prepared polymers showed larger molecular structure as compared with the LL. The FT-IR data imply that the ester group was established based on the hydroxyl group of lignin. In the 2D-HSQC spectra, the signals of 8HQ and hexadecyl could be clearly observed, suggesting that 8HQ was successfully introduced into the structure of lignin through fabricating ester groups. Furthermore, the SD of 8HQ was tested by a hydrolysis method, and it could reach 46.6%. Importantly, the synthesized lignin-based polymers showed an outstanding pH-sensitivity and a sustained release performance. The wettability could change with the pH, suggesting an outstanding pH-responsive performance. The example polymer L-Na showed about a 60 times slower release rate than the PM. Furthermore, the release performance is related to the pH of the solution. The releasing amount of 8HQ under an alkaline condition was higher than in an acidic condition. This work could provide a new method for the preparation of pH-responsive lignin-based polymers and for the sustained release of 8HQ.

## Figures and Tables

**Table 1:** Reaction conditions for each sample ^a^.

Entry	Sample	8HQ (Eq)	Alkali	Solvent	Temperature (°C)
1	L-60	2	NaOH	DMF	60
2	L-40	2	NaOH	DMF	40
3	L-80	2	NaOH	DMF	80
4	L-Et	2	Et_3_N	DMF	60
5	L-Na	2	Na_2_CO_3_	DMF	60
6	L-THF	2	NaOH	THF	60

^a^ The dosages of lignin and adipoyl chloride are 1.0 and 2.14 g, respectively.

**Table 2:** Hydroxyl content, 8HQ content, and degree of substitution in each sample.

Samples	^1^H NMR		Alkaline-Heating Method	
	Hydroxyl Content (mmol/g)	Decrease of Hydroxyl Content (%)	8HQ Content (mmol/g) ^a^	SD of 8HQ ^b^
LL	4.3	-	-	-
L-60	3.7	14.0%	1.4	30.0%
L-40	2.4	44.2%	1.4	31.9%
L-80	2.3	46.5%	1.5	32.5%
L-Et	2.2	48.8%	1.5	33.0%
L-Na	2.6	39.5%	2.1 (1.6) ^c^	46.6% (34.7%) ^c^
L-THF	0.6	86.0%	1	22.7%

^a^ The 8HQ content was calculated according to the equation: 8HQ content = n(8HQ)/m, where n(8HQ) is the amount (mmol) of the released 8HQ and m is the mass of the lignin-based polymer. ^b^ The SD of 8HQ was calculated according to the equation: SD = n(8HQ)/n(OH), where n(OH) is the hydroxyl content (mmol) of the added lignin. ^c^ The values in brackets are the 8HQ content and the SD of 8HQ, respectively, of PL-Na, which was prepared by purifying the L-Na via the dialysis method.

## Data Availability

Not applicable.
